# The prevalence of food allergy in cesarean-born children aged 0–3 years: A systematic review and meta-analysis of cohort studies

**DOI:** 10.3389/fped.2022.1044954

**Published:** 2023-01-17

**Authors:** Xiaoxian Yang, Chuhui Zhou, Chentao Guo, Jie Wang, Innie Chen, Shi Wu Wen, Daniel Krewski, Liqun Yue, Ri-hua Xie

**Affiliations:** ^1^ School of Health and Nursing, Wuxi Taihu University, Wuxi, China; ^2^ Affiliated Foshan Maternity & Child Healthcare Hospital, Southern Medical University, Foshan, China; ^3^ School of Nursing, Southern Medical University, Guangzhou, China; ^4^ Department of Epidemiology, Xishan Center for Disease Control and Prevention, Wuxi, China; ^5^ Department of Obstetrics & Gynecology, Faculty of Medicine, University of Ottawa, Ottawa, ON, Canada; ^6^ School of Epidemiology and Public Health, University of Ottawa Faculty of Medicine, Ottawa, ON, Canada; ^7^ Ottawa Hospital Research Institute, Ottawa, ON, Canada; ^8^ McLaughlin Centre for Population Health Risk Assessment, University of Ottawa Faculty of Medicine, Ottawa, ON, Canada; ^9^ Risk Science International, Ottawa, ON, Canada; ^10^ Department of Nursing, The Affiliated Hospital of Guangdong Medical University, Zhanjiang, China; ^11^ The Telfer School of Management, University of Ottawa, Ottawa, ON, Canada

**Keywords:** food allergy, cesarean-born, children aged 0–3 years, meta-analysis, prevalence of food allergy

## Abstract

**Purpose:**

Previous studies reported a higher risk of food allergy for cesarean-born children than vaginal-born children. This study aims to systematically compare the prevalence of food allergy among cesarean-born and vaginal-born children aged 0–3 years.

**Methods:**

Three English and two Chinese databases were searched using terms related to food allergies and cesarean sections. Cohort studies that reported the prevalence of food allergy in cesarean-born and vaginal-born children aged 0–3 years were included. Two reviewers performed study selection, quality assessment, and data extraction. The pooled prevalence of food allergy in cesarean-born and vaginal-born children was compared by meta-analysis.

**Results:**

Nine eligible studies, with 9,650 cesarean-born children and 20,418 vaginal-born children aged 0–3 years, were included. Of them, 645 cesarean-born children and 991 vaginal-born children were identified as having food allergies. The pooled prevalence of food allergy was higher in cesarean-born children (7.8%) than in vaginal-born children (5.9%). Cesarean section was associated with an increased risk of food allergy [odds ratio (OR): 1.45; 95% confidence interval (CI): 1.03–2.05] and cow's milk allergy (OR: 3.31; 95% CI: 1.98–5.53). Additionally, cesarean-born children with a parental history of allergy had an increased risk of food allergy (OR: 2.60; 95% CI: 1.28–5.27).

**Conclusion:**

This study suggests that cesarean sections was associated with an increased risk of food and cow's milk allergies in children aged 0–3 years. Cesarean-born children with a parental history of allergy demonstrated a higher risk for food allergy than did vaginal-born children. These results indicate that caregivers should be aware of the risks of food allergies in cesarean-born children, reducing the risk of potentially fatal allergic events. Further research is needed to identify the specific factors affecting food allergies in young children.

**Systematic Review Registration:**

http://www.crd.york.ac.uk/prospero, identifier: International Prospective Register of Systematic Reviews (NO. CRD42019140748).

## Introduction

Food allergy, defined as an adverse immune response to food proteins (1–3), is an important public health problem (4) that is becoming increasingly prevalent (2, 5). Specific foods that have been associated with allergic reactions include cow's milk, egg, wheat, soy, peanut, tree nuts, fish, and shellfish (6). At this time, the reasons for the apparent increase in food allergies in children are unclear (7).

The rate of cesarean section (CS) has been increasing in recent decades (2, 5). CS has been associated with an increased risk of developing asthma (8), allergic rhinitis (9), and other immune disorders in offspring (10). Although neonates acquire maternal vaginal and fecal microbiota during labor and delivery (11), CS interrupts this transfer process, thereby altering bacterial colonization of the gut (12). The altered gut ﬂora of cesarean-born children has been shown to prolong immunological immaturity and thereby increase the risk of allergic diseases (13).

Previous studies that assessed the association between mode of delivery and food allergy have yielded inconsistent results (7, 14–16). One study revealed that CS might be a risk factor for food allergens up to the age of 2 years (effect estimates for allergic sensitization against food allergens [odds ratio (OR) = 1.64 (1.03–2.63)] (14), whereas other studies found no convincing evidence that CS increased the risk of developing allergic diseases in children (15, 16). A systematic review synthesized the available evidence on the association between mode of delivery and food allergy and revealed that cesarean-born children under 10 years of age might have a higher risk of food allergy than vaginal-born children (7). However, no systematic review has estimated the prevalence of food allergy from different sources of foods among cesarean-born and vaginal-born children. This study aims to quantify and compare the prevalence of food allergy for cesarean-born and vaginal-born children aged 0–3 years through a systematic review and meta-analysis.

## Methods

This systematic review was conducted following the Preferred Reporting Items for Systematic Reviews and Meta-analyses (PRISMA) statement (17). The protocol was registered with the International Prospective Register of Systematic Reviews (NO. CRD42019140748) at http://www.crd.york.ac.uk/prospero (18).

### Search strategy

With the support of two research librarians with expertise in systematic reviews in health services, a systematic search of the literature was conducted to identify relevant studies using EMBASE, MEDLINE, Web of Science, CNKI, and Wanfang. The literature search included all published articles from inception to May 31, 2022. A combination of key terms and/or subject headings was applied, including food allergy terms (food hypersensitivity or (food or egg or nut or nuts* or peanut* or cashew or pistachio or hazelnut* or almond* or fish or soy or legume* or kiwi or apple or fruit or peach or milk or dairy or shellfish or wheat) or (allerg* or hypersensitivit*)) and cesarean section-related terms (c section* or Cesarean* or Caesarean*) (Supplementary Table S1). The databases were then combined and searched using the Covidence (a web-based software platform).

### Study selection

Titles and abstracts of articles retrieved by searching the five electronic databases were screened independently by two reviewers (XY and CG) to determine if they satisfied the predetermined inclusion/exclusion criteria. Fulltext screening was then performed to identify studies included in the systematic review. Disagreements regarding eligibility were resolved by discussion with the third reviewer (R-hX).

### Inclusion/exclusion criteria

Studies were included in this systematic review, if they: (1) targeted cesarean-born and vaginal-born children aged 0–3 years; (2) identified food allergy based on either self-report questionnaires (i.e., participants or their parents reported that they had any of the outcomes or not), or objective methods [i.e., skin prick test (SPT), specific immunoglobulin E (IgE), open food challenge (OFC)/double-blind placebo-controlled food challenge (DBPCFC), or convincing clinical history (i.e., outcomes confirmed by a physician)] (19); (3) had information about the sample size and prevalence of food allergy among cesarean-born and vaginal-born children aged 0–3 years; and (4) were published in English or Chinese.

Although food allergies typically appear around 1 year of age, there may be delays in identifying and reporting. By expanding the age range to include children up to 3 years old, we expected to achieve a more complete ascertainment of food allergies in young children. Due to the pre-specified age range in this study, with cesarean- and vaginal-born infants assessed in the same fashion; we did not expect bias to enter our review. Studies were excluded if they were abstracts, interviews, commentaries, or reviews. In addition, studies that did not report the prevalence of food allergy in children aged 0–3 were excluded.

### Data extraction

Two reviewers (XY and CZ) independently extracted relevant data from eligible studies using a standard form, including: the last name of the first author, publication year, country of origin, age of children, the identified methods of food allergy, parental history of food allergy, the study period, the number of cesarean-born and vaginal-born children, and the number of food, cow's milk, and egg allergies occurring in cesarean-born, and vaginal-born children. Any disagreements between the two reviewers were resolved by discussion with the third reviewer (R-hX).

### Quality assessment

The risk of bias and quality of the study were assessed by two reviewers (CG and CZ) independently using the Joanna Briggs Institute Meta-Analysis of Statistics Assessment and Review Instrument (JBI-MAStARI) (Supplementary Table S2). Any disagreements with respect to validity were resolved by the third reviewer (LY).

### Data analysis

Data analysis was conducted in the “meta” and “metaphor” modules using R software (version 3.6.2) (19). Heterogeneity across studies was assessed using the Higgins *I*^2^ statistic (20). In cases of significant heterogeneity (*I*^2 ^ >  0.5), the random effects model of Der Simonian and Laird (20) was used to obtain the prevalence of food allergy; otherwise, in cases of no inconsistency in the risk estimate (*I*^2^ < 0.5), a fixed effects model was used. When significant heterogeneity was observed, mixed-model meta-regression analysis was also conducted to explore the influence of potential moderators of heterogeneity using the restricted maximum-likelihood method. Subgroup analyses were conducted in terms of parental history of food allergy and identified methods of food allergy. Influence analysis was performed by serially removing each study one by one and excluding low-quality studies to examine their influence on the strength and stability of the pooled results. Potential publication biases were assessed graphically using funnel plots and statistically significance with *P* < 0.05 using Egger's tests (21).

## Results

### Search results

**Figure 1:**
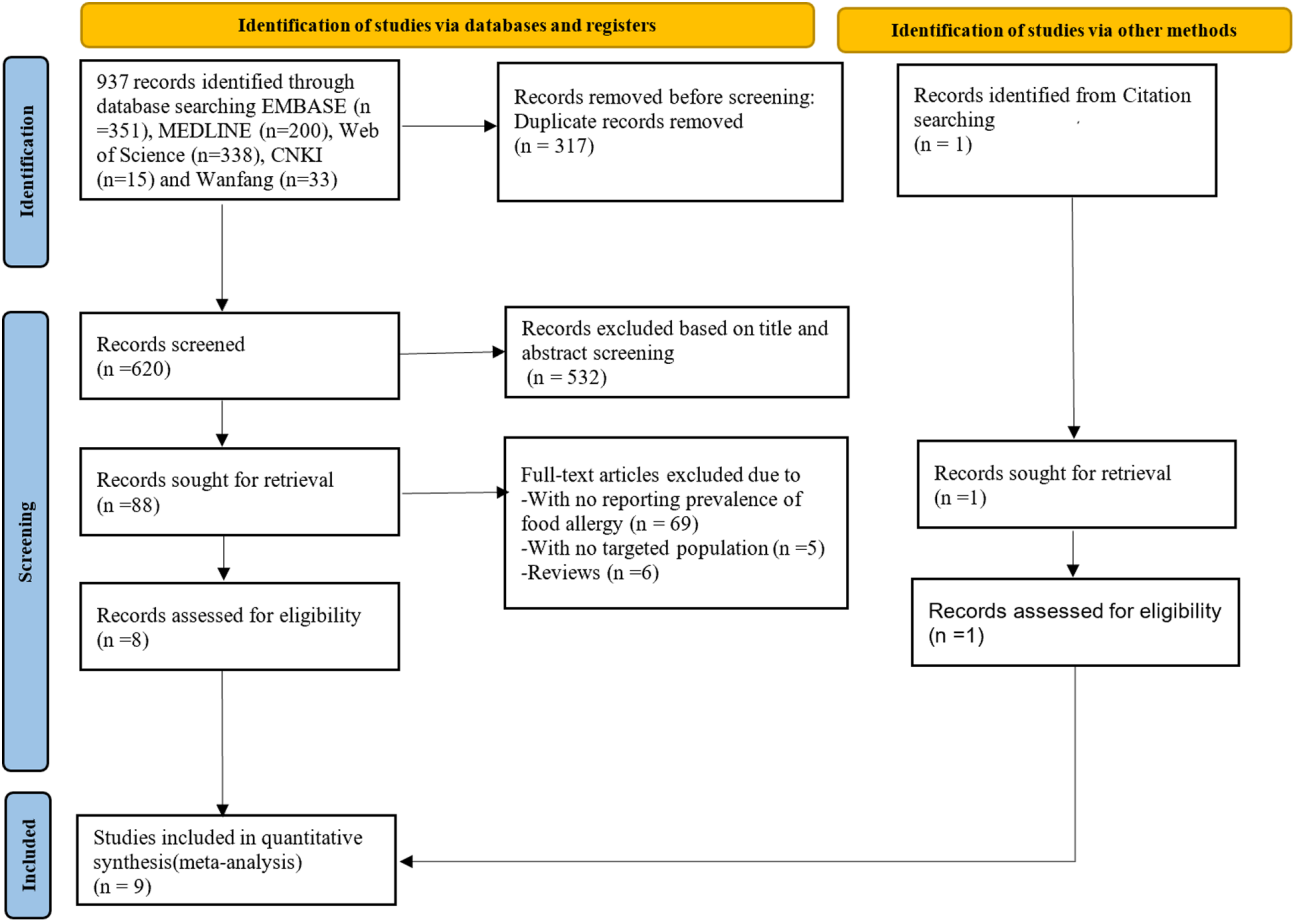
PRISMA flowchart showing selection of studies for review. PRISMA, Preferred Reporting Items for Systematic Reviews and Meta-analyses.

A total of 937 studies were identified through the searching of the five electronic databases. After removing duplicates and title/abstract screening for eligibility, 88 studies were selected for full text review. Of which, 80 were excluded because of their inability to satisfy the inclusion and exclusion criteria; and one study identified through citation searching was included. Finally, nine eligible studies (14, 16, 22–28) were chosen for data analysis (Figure 1).

### Characteristics of selected studies

The nine eligible studies (14, 16, 22–28) included in this review were published between 2003 and 2022 in seven countries: Norway, Germany, Australia, Sweden, China, and America. Their sample sizes ranged from 104 to 2,921, with a total of 9,650 cesarean-born children and 20,418 vaginal-born children aged 0–3 years. Of them, 645 cesarean-born children and 991 vaginal-born children had food allergies. Three studies (22, 23, 25) used exclusively perceived parental reactions to identify food allergy, and six (14, 16, 24, 26–28) used objective methods [SPT, IgE, OFC/DBPCFC, or clinical diagnosis (i.e., outcomes confirmed by a physician)]. Two studies (24, 25) recruited children whose parents had a history of allergy. Six studies (14, 16, 22–24, 26) reported the prevalence of any food allergy; three studies (23, 24, 28) reported cow's milk allergy; and two studies (23, 27) reported egg allergy in cesarean-born and vaginal-born children (Table 1).

**Table 1:** Characteristics of the nine included studies.

Author (year)	Country	Age of children (month)	Identified methods of allergy	Parental history of allergy	Study period	CS (*n*)	CS FA cases			VD (*n*)	VD FA cases		
							FA	CM	Egg		FA	CM	Egg
Eggesbø et al. (2003) (23)	Norway	0–24	Parental perceived reactions	N/A	1992–1995	328	9	NI	4	2,475	22	NI	17
Laubereau et al. (2004) (24)	Germany	0–12	Specific IgE test	Yes	1995–1998	104	18	9	NI	520	48	22	NI
Negele et al. (2004) (14)	Germany	0–24	Specific IgE test	N/A	1997–1999	348	39	NI	NI	1,681	153	NI	NI
Eggesbø et al. (2005) (25)	Norway	0–24	Parental perceived reactions	Yes	N/A	299	NI	5	NI	1,875	NI	7	NI
Kvenshagen et al. (2009) (26)	Norway	0–24	SPT + positive food challenge test	N/A	N/A	171	13	NI	NI	341	22	NI	NI
Koplin et al. (2012) (27)	Australia	11–15	SPT	N/A	2007–2011	1,641	NI	NI	136	3,292	NI	NI	316
Yang et al. (2018) (28)	China	0–12	Specific IgE test + positive food challenge test	N/A	2014–2015	2,355	NI	102	NI	4,413	NI	80	NI
Adeyeye et al. (2019) (22)	America	0–36	Parental perceived reactions	N/A	2008–2010	2,921	413	NI	NI	2,832	413	NI	NI
Currell et al. (2022) (16)	Australia	11–15	SPT + specific IgE test	N/A	2007–2011	1,483	153	NI	NI	2,989	333	NI	NI

N/A, not available; FA, food allergy; CM, cow's milk; CS, cesarean section; VD, vaginal delivery; NI, no information; IgE, immunoglobulin E; SPT, skin prick test.

### Methodological quality

Table 2 presents the results of the quality assessment of the nine studies (14, 16, 22–28) using the JBI-MAStARI quality scoring tool for cohort studies. The quality of these nine studies was generally high.

**Table 2:** Methodological qualities of nine included studies based on JBI-MAStARI.

Study	Q1	Q2	Q3	Q4	Q5	Q6	Q7	Q8	Q9
Eggesbø et al. (23)	Y	Y	Y	Y	Y	Y	Y	Y	Y
Laubereau et al. (24)	Y	Y	Y	Y	Y	Y	Y	Y	Y
Negele et al. (14)	Y	Y	Y	Y	Y	Y	Y	Y	Y
Eggesbø et al. (25)	Y	Y	Y	Y	Y	Y	Y	Y	Y
Kvenshagen et al. (26)	Y	Y	Y	Y	Y	Y	Y	Y	Y
Koplin et al. (27)	Y	Y	Y	Y	Y	Y	Y	Y	Y
Yang et al. (28)	Y	Y	Y	Y	Y	Y	Y	Y	Y
Adeyeye et al. (22)	Y	Y	Y	Y	Y	Y	Y	Y	Y
Currell et al. (16)	Y	Y	Y	Y	Y	Y	Y	Y	Y
Overall score (%)	Y	Y	Y	Y	Y	Y	Y	Y	Y

JBI-MAStARI, Joanna Briggs Institute Meta-Analysis of Statistics Assessment and Review Instrument; Y, yes; N, no; U, unclear; NA, not applicable; Q1–Q9 (Supplementary Table S2).

### The pooled prevalence of food allergy for cesarean-born and vaginal-born children

**Figure 2:**
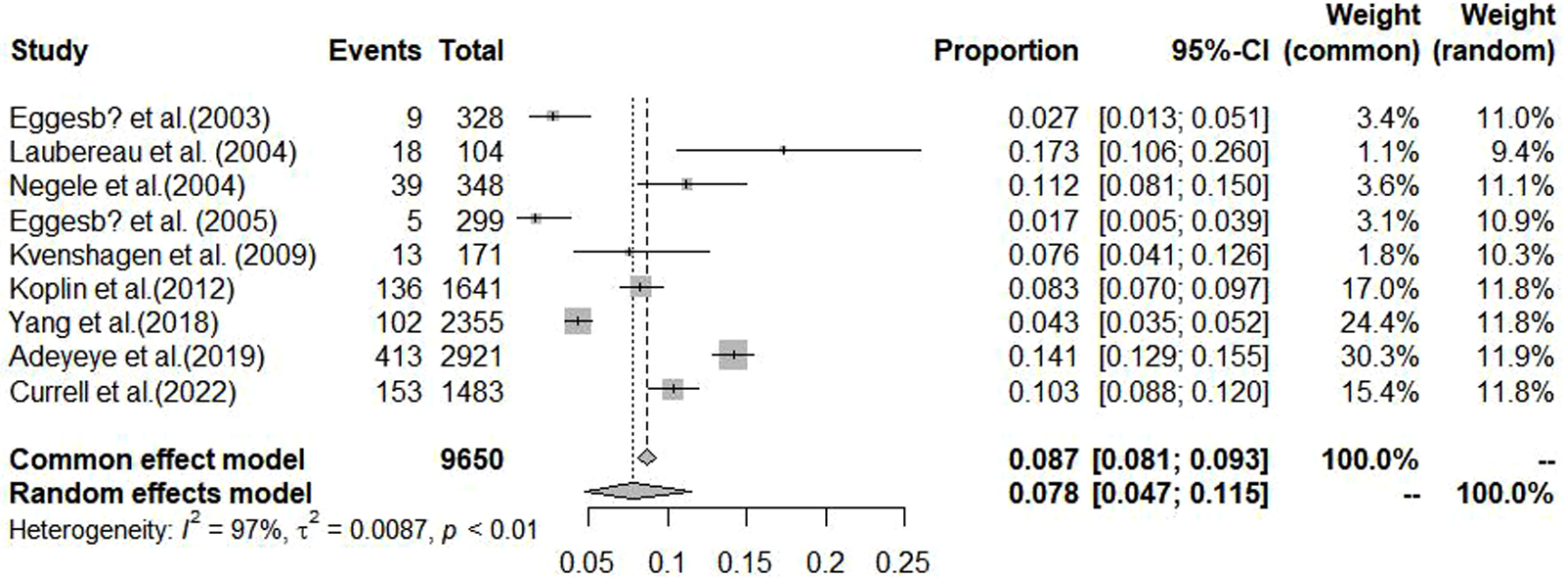
Pooled prevalence of food allergy among cesarean-born children aged 0–3 years.

**Figure 3:**
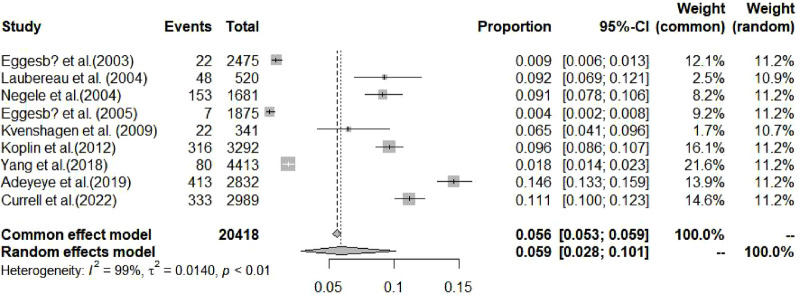
Pooled prevalence of food allergy among vaginal-born children aged 0–3 years.

**Figure 4:**
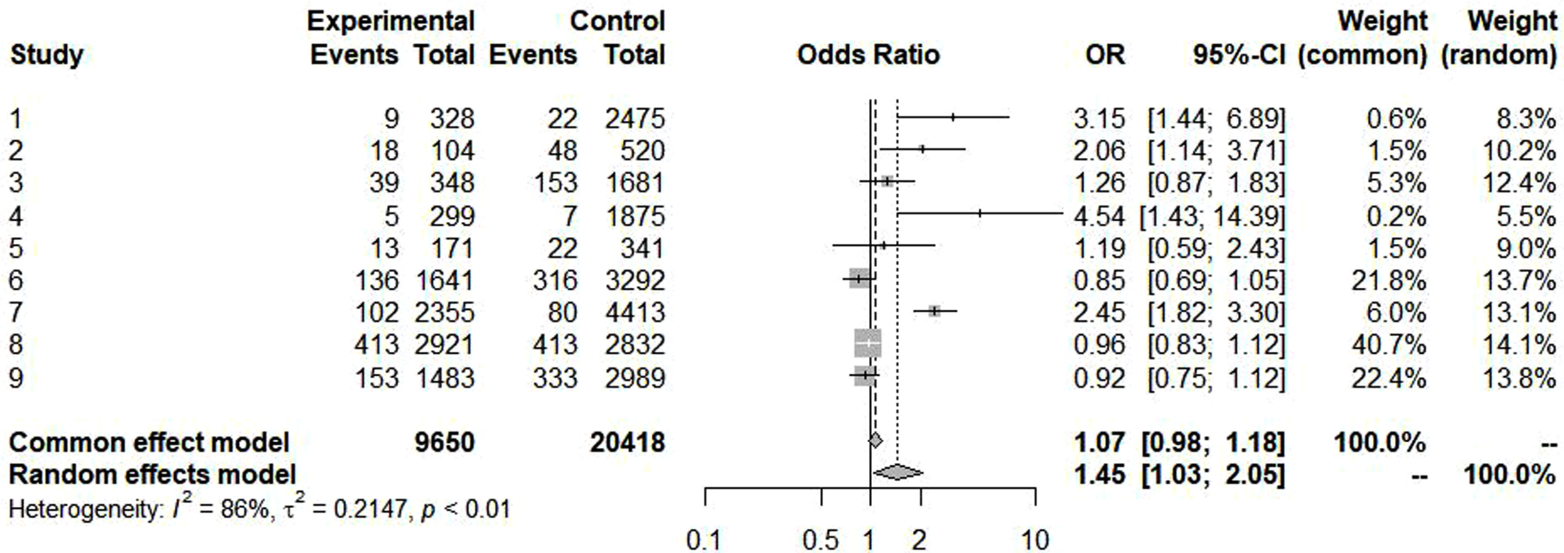
Association between cesarean section and food allergy in children aged 0–3 years.

Figures 2, 3 summarize the pooled prevalence of food allergy of cesarean-born and vaginal-born children. The pooled prevalence of food allergy was 7.8% [95% confidence interval (CI): 4.7%–11.5%, *I*^2^ = 96.6%, 95% CI: 95.1%–97.7%] in cesarean-born children (Figure 2) and 5.9% (95% CI: 2.8%–10.1%; *I*^2 ^= 99.3%, 95% CI: 99.1%–99.4%) in vaginal-born children (Figure 3). Overall, CS was associated with an increased risk of food allergy (OR: 1.45, 95% CI: 1.03–2.05; *I*^2 ^= 86.0%, 95% CI: 75.5–92.1) (Figure 4).

### Subgroup analysis

Table 3 presents results from our subgroup analysis. The prevalence of food allergy was higher in cesarean-born children than in vaginal-born children aged 0–3 years according to identified methods of food allergy, type of food allergies, parental history of allergy, age group and country. In addition, cesarean-born children with cow's milk allergy (OR: 3.31; 95% CI: 1.98–5.53) and with a parental history of allergy (OR: 2.60; 95% CI: 1.28–5.27), had a higher risk of food allergy than did vaginal-born children.

**Table 3:** Subgroup analysis of the pooled prevalence of food allergy among children aged 0–3 years.

Subgroup	Cesarean section					Vaginal delivery					
	Studies (*n*)	FA (*n*)	*n*	Pooled prevalence (%, 95% CI)	*P*	Studies (*n*)	FA (*n*)	*n*	Pooled prevalence (%, 95% CI)	*P*	OR
Identified methods of food allergy											
Parental perceived	3	427	3,548	5.2 (0.5–14.3)	0.37	3	442	7,182	3.3 (0.00–14.4)	0.39	2.13 (0.80–5.64)
Objective measures	6	461	6,102	9.1 (6.2–12.4)		6	952	13,236	7.5 (4.6–11.0)		1.32 (0.91–1.90)
Type of food allergy											
FA	6	645	5,355	9.9 (6.1–14.6)	<0.01	6	991	10,838	7.8 (3.9–12.8)	<0.01	1.29 (0.93–1.79)
CM	3	116	2,758	6.2 (0.7–16.8)		3	109	6,808	1.8 (0.3–4.5)		3.31 (1.98–5.53)
Egg	2	140	1,969	5.3 (1.2–12.1)		2	333	5,767	3.9 (0.00–17.1)		1.77 (0.38–8.19)
Parental history of allergy											
Yes	2	23	403	8.0 (5.2–11.4)	0.95	2	55	2,395	3.4 (0.00–17.1)	0.55	2.60 (1.28–5.27)
No	7	865	9,247	7.5 (0.00–29.1)		7	1,339	18,023	6.8 (3.2–11.4)		1.29 (0.91–1.83)
Children's age											
0–12	2	120	2,459	9.5 (1.0–25.5)	0.01	2	128	4,933	4.8 (0.3–14.5)	<0.01	2.37 (1.82–3.09)
0–24	6	355	4,270	6.5 (3.5–10.3)		6	853	12,653	5.1 (1.7–10.3)		1.36 (0.87–2.13)
0–36	1	413	2,921	14.1 (12.9–15.5)		1	413	2,832	14.6 (13.3–15.9)		2.37 (1.82–3.09)
Country											
China	1	102	2,355	4.3 (3.5–5.2)	0.02	1	80	4,413	1.8 (1.4–2.3)	<0.01	1.32 (0.94–1.84)
Other countries	8	786	7,295	8.3 (4.9–12.5)		8	1,314	16,005	6.6 (3.1–11.2)		2.45 (1.82–3.30)

P, P-value of test of difference within each subgroup; FA, food allergy; CM, cow's milk; CI, confidence interval.

### Influence analysis and publication bias

**Figure 5:**
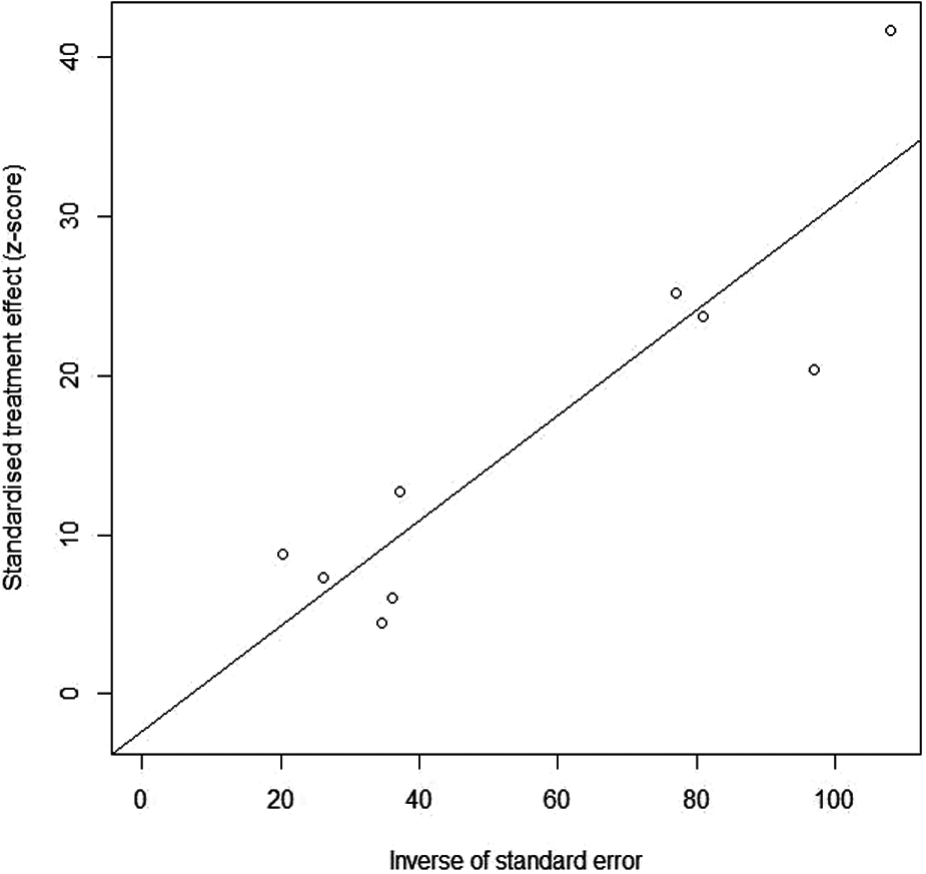
Egger's funnel plot of the nine included studies.

Influence analysis based on one-by-one removal of the nine eligible studies (14, 16, 22–28) indicated that the pooled prevalence of food allergy in cesarean-born children varied from 7.1% (95% CI: 6.5%–7.7%) to 10.8% (95% CI:10.1%–11.5%), with the corresponding *I*^2^ statistic ranging from 91.8% to 96.1%, while the pooled prevalence of food allergy in vaginal-born children varied from 5.6% (95% CI: 5.3%–5.9%) to 8.2% (95% CI: 7.8%–8.7%), with the corresponding *I*^2^ statistic varying from 97.6% to 98.7% (Table 4). Although the number of the studies included in the final analysis was less than 10, the funnel plot was used to evaluate their potential publication bias (Figure 5), providing no evidence of potential publication bias, with *P*-value for the Egger's rank test being 0.6.

**Table 4:** Influence analysis of the nine eligible studies.

Study	Cesarean section			Vaginal delivery		
	Pooled prevalence (%)	95% CI (%)	*I*^2^ (%)	Pooled prevalence (%)	95% CI (%)	*I*^2^ (%)
Omitting Eggesbø et al. (23)	9.43	8.85–10.04	95.7	7.65	7.27–8.04	98.3
Omitting Laubereau et al. (24)	9.91	8.55–9.71	96.0	6.76	6.42–7.12	98.7
Omitting Negele et al. (14)	9.13	8.56–9.73	96.1	6.62	6.28–6.99	98.7
Omitting Eggesbø et al. (25)	9.44	8.87–10.05	95.7	7.48	7.11–7.87	98.5
Omitting Kvenshagen et al. (26)	9.23	8.66–9.83	96.1	6.83	6.49–7.19	98.7
Omitting Koplin et al. (27)	9.39	8.77–10.05	95.9	6.29	5.94–6.67	98.7
Omitting Yang et al. (28)	10.77	10.08–11.51	91.8	8.21	7.79–8.65	97.6
Omitting Adeyeye et al. (22)	7.06	6.47–7.70	92.4	5.58	5.25–5.93	98.4
Omitting Currell et al. (16)	9.00	8.40–9.64	96.1	6.09	5.74–6.45	98.7

CI, confidence interval.

### Meta-regression analysis

Meta-regression analysis revealed that the identified methods of food allergy and the infants' age were the significant sources of heterogeneity in estimates of the prevalence of food allergy (*P *< 0.05) (Table 5).

**Table 5:** Meta-regression analyses of the effects of potential modifying factors of the prevalence of food allergy in children.

	No. of studies	Cesarean section				Vaginal delivery			
		Estimate	Standard error	*Z*-value	*P*-value	Estimate	Standard error	*Z*-value	*P*-value
Identified methods of food allergies	9	1.6711	0.5548	3.0119	0.0026	2.8570	0.7481	3.8191	0.0001
Type of food allergies	9	−0.3700	0.2119	−1.7465	0.0807	−0.4098	0.3339	−1.2271	0.2198
Parental history of allergy	9	0.8918	0.6577	1.3558	0.1752	0.6929	0.8962	0.7732	0.4394
Age	9	1.2916	0.6211	2.0796	0.0376	2.0614	0.8697	2.3702	0.0178
Country	9	0.4890	0.7654	0.6389	0.5229	0.3506	1.1407	0.3073	0.7586

## Discussion

### Main findings of the study

Meta-analysis of the nine studies included in our systematic review found that the pooled prevalence of food allergy was 7.8% in cesarean-born children, which was higher than 5.9% in vaginal-born children, with an OR = 1.45 (95% CI: 1.03–2.05). Cesarean section was associated with an increased risk of both food allergy and cow's milk allergy in children. Cesarean-born children aged 0–3 years with a parental history of allergy also developed food allergies at a higher rate than vaginal-born children of the same age (OR: 2.60; 95% CI: 1.28–5.27).

### Strengths and limitations

To the best of our knowledge, this is the first systematic review/meta-analysis that compared the pooled prevalence of food allergy from various sources of foods for cesarean-born and vaginal-born children aged 0–3 years. Our consistent findings from the subgroup, influence analyses and meta-regression analysis indicated the robustness of the results. Also, we did not find any publication bias.

Several limitations of this study must be acknowledged. First, although the overall sample size was large, the small sample size for specific food allergies complicated the interpretation of these results. For example, only two studies (23, 27) included children who were allergic to eggs. Second, heterogeneities have been partly explained by our subgroup analyses or meta-regressions. Our influence analysis showed considerable variations in the results from one-by-one removal and the potential existence of influential cases/outliers. Thus, we could not adjust for potential confounders in the meta-analysis due to heterogeneity for specific foods, including important sources of food allergies such as peanuts, fish, and wheat, as this information was not available in the original studies. Third, the original studies lacked information regarding breastfeeding, formula feeding, eczema, and the timing to take complementary foods, precluding an analysis of the potential influence of these factors on our meta-analytic results.

### Interpretation

Sensitization to food allergens during early childhood could be an important predictor for developing allergic airway diseases later in childhood (29). Our study found that more than 7.8% of cesarean-born children aged 0–3 years were allergic to any food sources, higher than the rate of 5.9% of vaginal-born children and higher than the 6% reported in children aged 0–3 years observed in another systematic review in the general population regardless of mode of delivery (30). These results support our hypothesis that CS may increase the risk of developing food allergies in children (31).

One possible explanation for the increased prevalence of food allergy in cesarean-born children aged 0–3 years is that their gut microbiota are different from those of vaginal-born children (15, 32). The intestinal microbiota and early-life microbial exposure play important roles in the development of the immune system (32), as well as in educating the immune system (33). CS could lead to delayed bacterial colonization of the gut because it limited newborn exposure to maternal vaginal and fecal microflora (34). The interruption of natural colonization has been speculated to derive from the altered immune development as well as allergic diseases among children (34). Previous studies demonstrated that mode of delivery is an imperative independent factor impacting natural colonization, especially in the first months of life (35–37). Thus, CS may be associated with an increased risk of immune and metabolic disorders, as compared with vaginal delivery (VD) (38). However, some studies showed conflicting results, namely, that CS was not associated with food allergy risk in infants (15, 16). These might be because there are diverse ways to distinguish whether an elective or emergency cesarean is with or without labor and to measure food allergy, leading to contradicting results (16).

Cow's milk protein allergy (CMPA) is considered the most common food allergy in infants (39). The subgroup analyses conducted in the present study suggest that cesarean-born children have a higher risk of cow's milk allergy than in vaginal-born children. The underlying mechanism is unknown but may be related to the gut microbiota composition in feces from children with food allergies (28, 40) which have higher proportions of the *Clostridium coccoides* group and *Atopobium* cluster as well as a higher proportion of the (sum of) different bacterial groups in comparison to healthy infant feces (40). It has also been shown that bacterial colonization of the gut in cesarean-born children aged 0–3 years differs from that in vaginal-born children (24).

The pooled prevalence of food allergy in cesarean-born children aged 0–3 years whose parents had a history of allergy was higher than that in vaginal-born infants, consistent with a previous study (7). Genetic predisposition seems to be the biggest risk factor for allergic diseases (41). Currently, it is widely accepted that children with a family history of “allergy” are at generally increased risk of food allergy (42). A family study has shown that food sensitization and allergy are more common in those with a first degree relative with food allergy (43). A study in Korea has demonstrated maternal allergy is associated with an increased risk of parent-reported food allergy in children's first year of life (44). However, genetics alone cannot explain the rising global prevalence of food allergy (41); CS and parental history of allergy may have cooperative effects on food allergy in children.

## Conclusion

Cesarean section was associated with an increased risk of food allergy and cow's milk allergy in cesarean-born children aged 0–3. Cesarean-born children with a parental history of allergy developed a higher risk for food allergies than did vaginal-born children. These results indicate that caregivers should be aware of the risks of food allergy in cesarean-born children, reducing the risk of potentially fatal allergic events. Further research is needed to identify the specific factors affecting food allergies in young children.

## Data Availability

The original contributions presented in the study are included in the article/Supplementary Material, further inquiries can be directed to the corresponding authors.

## References

[1] Sackeyfio A Senthinathan A Kandaswamy P Barry PW Shaw B Baker M Diagnosis and assessment of food allergy in children and young people: summary of NICE guidance. Br Med J. (2011) 342:d747. 10.1136/bmj.d747 21345912

[2] Peters RL Krawiec M Koplin JJ Santos AF. Update on food allergy. Pediatr Allergy Immunol. (2021) 32(4):647–57. 10.1111/pai.13443 33370488 PMC8247869

[3] Cianferoni A Spergel JM. Food allergy: review, classification and diagnosis. Allergol Int. (2009) 58(4):457–66. 10.2332/allergolint.09-RAI-0138 19847094

[4] Nwaru BI Hickstein L Panesar SS Muraro A Werfel T Cardona V The epidemiology of food allergy in Europe: a systematic review and meta-analysis. Allergy. (2014) 69(1):62–75. 10.1111/all.12305 24205824

[5] Nwaru BI Hickstein L Panesar SS Roberts G Muraro A Sheikh A Prevalence of common food allergies in Europe: a systematic review and meta-analysis. Allergy. (2014) 69(8):992–1007. 10.1111/all.12423 24816523

[6] Allen KJ Koplin JJ. The epidemiology of IgE-mediated food allergy and anaphylaxis. Immunol Allergy Clin North Am. (2012) 32(1):35–50. 10.1016/j.iac.2011.11.008 22244231

[7] Koplin J Allen K Gurrin L Osborne N Tang ML Dharmage S. Is caesarean delivery associated with sensitization to food allergens and IgE-mediated food allergy: a systematic review. Pediatr Allergy Immunol. (2008) 19(8):682–7. 10.1111/j.1399-3038.2008.00731.x 19076564

[8] Darabi B Rahmati S HafeziAhmadi MR Badfar G Azami M. The association between caesarean section and childhood asthma: an updated systematic review and meta-analysis. Allergy Asthma Clin Immunol. (2019) 15(1):1–13. 10.1186/s13223-019-0367-9 31687033 PMC6820931

[9] Pistiner M Gold DR Abdulkerim H Hoffman E Celedón JC. Birth by cesarean section, allergic rhinitis, and allergic sensitization among children with a parental history of atopy. J Allergy Clin Immunol. (2008) 122(2):274–9. 10.1016/j.jaci.2008.05.007 18571710 PMC4762591

[10] Kristensen K Henriksen L. Cesarean section and disease associated with immune function. J Allergy Clin Immunol. (2016) 137(2):587–90. 10.1016/j.jaci.2015.07.040 26371844

[11] Hyman RW Fukushima M Diamond L Kumm J Giudice LC Davis RW. Microbes on the human vaginal epithelium. Proc Natl Acad Sci U S A. (2005) 102(22):7952–7. 10.1073/pnas.0503236102 15911771 PMC1142396

[12] Loo EXL Sim JZT Loy SL Goh A Chan YH Tan KH Associations between caesarean delivery and allergic outcomes: results from the GUSTO study. Ann Allergy Asthma Immunol. (2017) 118(5):636–8. 10.1016/j.anai.2017.02.021 28477794 PMC5505471

[13] Ly NP Ruiz-Pérez B Onderdonk AB Tzianabos AO Litonjua AA Liang C Mode of delivery and cord blood cytokines: a birth cohort study. Clin Mol Allergy. (2006) 4(1):1–11. 10.1186/1476-7961-4-13 17002791 PMC1592116

[14] Negele K Heinrich J Borte M von Berg A Schaaf B Lehmann I Mode of delivery and development of atopic disease during the first 2 years of life. Pediatr Allergy Immunol. (2004) 15(1):48–54. 10.1046/j.0905-6157.2003.00101.x 14998382

[15] McKeever TM Lewis SA Smith C Hubbard R. Mode of delivery and risk of developing allergic disease. J Allergy Clin Immunol. (2002) 109(5):800–2. 10.1067/mai.2002.124046 11994703

[16] Currell A Koplin JJ Lowe AJ Perrett KP Ponsonby AL Tang MLK Mode of birth is not associated with food allergy risk in infants. J Allergy Clin Immunol Pract. (2022) 10(8):2135–43.e3. 10.1016/j.jaip.2022.03.031 35597762

[17] Moher D Shamseer L Clarke M Ghersi D Liberati A Petticrew M Preferred reporting items for systematic review and meta-analysis protocols (PRIS MA-P) 2015 statement. Syst Rev. (2015) 4(1):1. 10.1186/2046-4053-4-1 25554246 PMC4320440

[18] PROSPERO. Xiaoxian Yang YC Rihua Xie SS. Wen: Prevalence of food allergy in cesarean-born infants: a systematic review (2019). Available at: https://wwwcrdyorkacuk/prospero/display_recordphp?ID=CRD42019140748. (Accessed September 18, 2019). 10.3389/fped.2022.1044954 PMC9887154 36733768

[19] Laia-Dias I Lozoya-Ibáñez C Skypala I Gama JMR Nurmatov U Lourenço O Prevalence and risk factors for food allergy in older people: protocol for a systematic review. BMJ Open. (2019) 9(8):e029633. 10.1136/bmjopen-2019-029633 31446411 PMC6719770

[20] Higgins JPT Green S, (editors). Cochrane handbook for systematic reviews of interventions, version 5.1.0 (updated March 2011). The Cochrane Collaboration (2011). Available at: www.cochrane-handbook.org.

[21] Higgins JP Thompson SG Deeks JJ Altman DG. Measuring inconsistency in meta-analyses. Br Med J. (2003) 327(7414):557–60. 10.1136/bmj.327.7414.557 12958120 PMC192859

[22] Adeyeye TE Yeung EH McLain AC Lin S Lawrence DA Bell EM. Wheeze and food allergies in children born via cesarean delivery: the upstate kids study. Am J Epidemiol. (2019) 188(2):355–62. 10.1093/aje/kwy257 30475936 PMC6357798

[23] Eggesbø M Botten G Stigum H Nafstad P Magnus P. Is delivery by cesarean section a risk factor for food allergy? J Allergy Clin Immunol. (2003) 112(2):420–6. 10.1067/mai.2003.1610 12897751

[24] Laubereau B Filipiak-Pittroff B von Berg A Grübl A Reinhardt D Wichmann HE Caesarean section and gastrointestinal symptoms, atopic dermatitis, and sensitisation during the first year of life. Arch Dis Child. (2004) 89(11):993–7. 10.1136/adc.2003.043265 15499049 PMC1719727

[25] Eggesbø M Botten G Stigum H Samuelsen SO Brunekreef B Magnus P. Cesarean delivery and cow milk allergy/intolerance. Allergy. (2005) 60(9):1172–3. 10.1111/j.1398-9995.2005.00857.x 16076303

[26] Kvenshagen B Halvorsen R Jacobsen M. Is there an increased frequency of food allergy in children delivered by caesarean section compared to those delivered vaginally? Acta Paediatr. (2009) 98(2):324–7. 10.1111/j.1651-2227.2008.01074.x 18976354

[27] Koplin JJ Dharmage SC Ponsonby AL Tang ML Lowe AJ Gurrin LC Environmental and demographic risk factors for egg allergy in a population-based study of infants. Allergy. (2012) 67(11):1415–22. 10.1111/all.12015 22957661

[28] Yang M Tan M Wu J Chen Z Long X Zeng Y Prevalence, characteristics, and outcome of cow's milk protein allergy in Chinese infants: a population-based survey. JPEN J Parenter Enteral Nutr. (2019) 43(6):803–8. 10.1002/jpen.1472 30452099

[29] Kulig M Bergmann R Tacke U Wahn U Guggenmoos-Holzmann I. Long-lasting sensitization to food during the first two years precedes allergic airway disease. The mas study group, Germany. Pediatr Allergy Immunol. (2010) 9(2):61–7. 10.1111/j.1399-3038.1998.tb00305.x 9677600

[30] Panel NSE Boyce JA Assa’ad A Burks AW Jones SM Sampson HA Guidelines for the diagnosis and management of food allergy in the United States: report of the NIAID-sponsored expert panel. J Allergy Clin Immunol. (2010) 126(6 Suppl):S1–58. 10.1016/j.jaci.2010.10.007 21134576 PMC4241964

[31] Sandall J Tribe RM Avery L Mola G Visser GH Homer CS Short-term and long-term effects of caesarean section on the health of women and children. Lancet. (2018) 392(10155):1349–57. 10.1016/S01406736(18)31930-5 30322585

[32] Zimmermann P Messina N Mohn WW Finlay BB Curtis N. Association between the intestinal microbiota and allergic sensitization, eczema, and asthma: a systematic review. J Allergy Clin Immunol. (2019) 143(2):467–85. 10.1016/j.jaci.2018.09.025 30600099

[33] Olszak T An D Zeissig S Vera MP Richter J Franke A Microbial exposure during early life has persistent effects on natural killer T cell function. Science. (2012) 336(6080):489–93. 10.1126/science.1219328 22442383 PMC3437652

[34] Gensollen T Iyer SS Kasper DL Blumberg RS. How colonization by microbiota in early life shapes the immune system. Science. (2016) 352(6285):539–44. 10.1126/science.aad9378 27126036 PMC5050524

[35] Stokholm J Thorsen J Chawes BL Schjørring S Krogfelt KA Bønnelykke K Cesarean section changes neonatal gut colonization. J Allergy Clin Immunol. (2016) 138(3):881–9.e2. 10.1016/j.jaci.2016.01.028 27045582

[36] Bokulich NA Chung J Battaglia T Henderson N Jay M Li H Antibiotics, birth mode, and diet shape microbiome maturation during early life. Sci Transl Med. (2016) 8(343):343ra82. 10.1126/scitranslmed.aad7121 27306664 PMC5308924

[37] Hoen AG Coker MO Madan JC Pathmasiri W McRitchie S Dade EF Association of cesarean delivery and formula supplementation with the stool metabolome of 6-week-old infants. Metabolites. (2021) 11(10):702. 10.3390/metabo11100702 34677417 PMC8540440

[38] Dominguez-Bello MG De Jesus-Laboy KM Shen N Cox LM Amir A Gonzalez A Partial restoration of the microbiota of cesarean-born infants via vaginal microbial transfer. Nat Med. (2016) 22(3):250–3. 10.1038/nm.4039 26828196 PMC5062956

[39] Vandenplas Y Benninga M Broekaert I Falconer J Gottrand F Guarino A Functional gastro-intestinal disorder algorithms focus on early recognition, parental reassurance and nutritional strategies. Acta Paediatr. (2016) 105(3):244–52. 10.1111/apa.13270 26584953

[40] Thompson-Chagoyan OC Fallani M Maldonado J Vieites JM Khanna S Edwards C Faecal microbiota and short-chain fatty acid levels in faeces from infants with cow's milk protein allergy. Int Arch Allergy Immunol. (2011) 156(3):325–32. 10.1159/000323893 21720179

[41] Yang HJ Lee SY Suh DI Shin YH Kim BJ Seo JH The Cohort for Childhood Origin of Asthma and allergic diseases (COCOA) study: design, rationale and methods. BMC Pulm Med. (2014) 14:109. 10.1186/1471-2466-14-109 24990471 PMC4099383

[42] Koplin JJ Allen KJ Gurrin LC Peters RL Lowe AJ Tang ML The impact of family history of allergy on risk of food allergy: a population-based study of infants. Int J Environ Res Public Health. (2013) 10(11):5364–77. 10.3390/ijerph10115364 24284354 PMC3863850

[43] Tsai HJ Kumar R Pongracic J Liu X Story R Yu Y Familial aggregation of food allergy and sensitization to food allergens: a family-based study. Clin Exp Allergy. (2009) 39(1):101–9. 10.1111/j.1365-2222.2008.03111 19016802 PMC2729087

[44] Kim J Chang E Han Y Ahn K Lee SI. The incidence and risk factors of immediate type food allergy during the first year of life in Korean infants: a birth cohort study. Pediatr Allergy Immunol. (2011) 22(7):715–9. 10.1111/j.1399-3038.2011.01163.x 21539613

