# Supplementary material for: The prevalence of food allergy in cesarean-born children aged 0–3 years: A systematic review and meta-analysis of cohort studies
Source: Front Pediatr. 2023 Jan 17;10:1044954. doi: 10.3389/fped.2022.1044954 (PMC9887154; doi:10.3389/fped.2022.1044954)
Supplement: Supplementary file 1 [file Table1.docx]

Supplementary Material

The prevalence of food allergy in cesarean-born children aged 0-3 years: a systematic review and meta-analysis of cohort studies

Xiaoxian Yang^1†^, Chuhui Zhou^2,3†^, Chentao Guo^4†^, Jie Wang^2,3^, Innie Chen^5-7^, Shi Wu Wen^5-7^, Daniel Krewski ^6,8,9^, Liqun Yue^10*^, Ri-hua Xie^2,11*^

*** Correspondence:**  Ri-hua Xie （E-mail address: [xierihua928@hotmail.com](mailto:xierihua928@hotmail.com)）

# Supplementary Tables

**Supplementary Table 1. Detailed Search Strategy**

| **Database** | Search Strategy |
| --- | --- |
| **EMBASE, MEDLINE** | 1 exp Cesarean Section/  2 (c section* or C?esarean*).tw,kw.  3 1 or 2  4 exp Food Hypersensitivity/  5 (food allerg* or food Hypersensitivit*).kw.  6 Food Intolerance/  7 ((food or egg or nut or nuts* or peanut* or hazelnut* or almond* or fish or soy or legume* or kiwi or apple or fruit or peach or milk or dairy or shellfish or wheat) adj3 (allerg* or hypersensitivit*)).tw.  8 (additive* adj (allerg* or hypersensitiv*)).tw.  9 4 or 5 or 6 or 7 or 8  10 3 and 9  11 10 use medall  12 exp cesarean section/  13 (c section* or C?esarean*).tw.  14 or/12-13  15 exp food allergy/  16 ((food or egg or nut or nuts* or peanut* or hazelnut* or almond* or fish or soy or legume* or kiwi or apple or fruit or peach or milk or dairy or shellfish or wheat) adj3 (allerg* or hypersensitivit*)).tw.  17 (additive* adj (allerg* or hypersensitiv*)).tw.  18 nutritional intolerance/  19 oral allergy syndrome/  20 or/15-19  21 14 and 20  22 21 use emczd  23 11 or 22  24 remove duplicates from 23  25 24 use medall  26 24 use emczd |
| **Web of Science** | # 3=#2 AND #1  Indexes=SCI-EXPANDED, CPCI-S, CPCI-SSH, ESCI  #2 =TOPIC: ("c section*") OR TOPIC: (cesarean) OR TOPIC: (caesarean)  Indexes=SCI-EXPANDED, CPCI-S, CPCI-SSH, ESCI  #1=TOPIC: ((food or egg or nut or nuts* or peanut* or hazelnut* or almond* or fish or soy or legume* or kiwi or apple or fruit or peach or milk or dairy or shellfish or wheat) NEAR/3 (allerg* or hypersensitivit*))  Indexes=SCI-EXPANDED, CPCI-S, CPCI-SSH, ESCI |
| **CNKI** | FT = ("食物过敏") * ("剖宫产" + "剖腹产" + "分娩方式") * ("婴幼儿"  + "幼儿" + "儿童") |
| **Wangfan** | (食物过敏) AND (剖宫产 OR 剖腹产 OR 分娩方式) AND (婴幼儿OR 幼儿OR 儿童) |

**Supplementary Table 2. JBI Critical Appraisal Checklist for Studies Reporting Prevalence Data**

Reviewer Date

Author Year Record Number

|  | Yes | No | Unclear | Not applicable |
| --- | --- | --- | --- | --- |
| Q1. Was the sample frame appropriate to address the target population? |  |  |  |  |
| Q2. Were study participants sampled in an appropriate way? |  |  |  |  |
| Q3. Was the sample size adequate? |  |  |  |  |
| Q4. Were the study subjects and the setting described in detail? |  |  |  |  |
| Q5. Was the data analysis conducted with sufficient coverage of the identified sample? |  |  |  |  |
| Q6. Were valid methods used for the identification of the condition? |  |  |  |  |
| Q7. Was the condition measured in a standard, reliable way for all participants? |  |  |  |  |
| Q8. Was there appropriate statistical analysis? |  |  |  |  |
| Q9. Was the response rate adequate, and if not, was the low response rate managed appropriately? |  |  |  |  |
| Overall appraisal: □ Include □ Exclude □ Seek further info | | | | |
| Comments (Including reason for exclusion) | | | | |
