# Supplementary figures and images for: The prevalence of food allergy in cesarean-born children aged 0–3 years: A systematic review and meta-analysis of cohort studies
Source: Front Pediatr. 2023 Jan 17;10:1044954. doi: 10.3389/fped.2022.1044954 (PMC9887154; doi:10.3389/fped.2022.1044954)

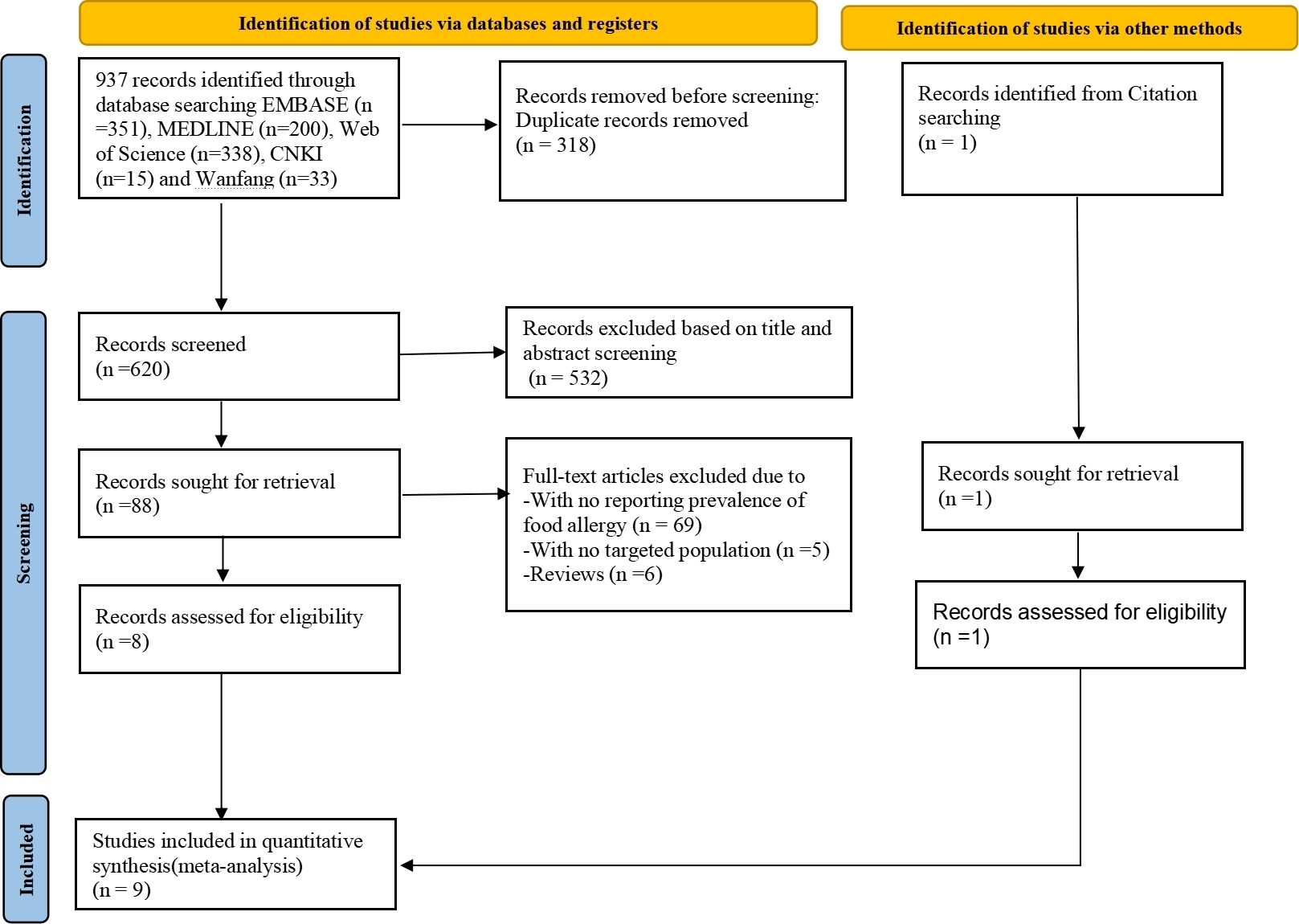

Supplement: Supplementary file 2 [file Image1.jpeg]
